# Nonlinear wavefront engineering with metasurface decorated quartz crystal

**DOI:** 10.1515/nanoph-2021-0464

**Published:** 2021-11-02

**Authors:** Ningbin Mao, Yutao Tang, Mingke Jin, Guanqing Zhang, Yang Li, Xuecai Zhang, Zixian Hu, Wenhao Tang, Yu Chen, Xuan Liu, Kingfai Li, Kokwai Cheah, Guixin Li

**Affiliations:** Department of Materials Science and Engineering, Southern University of Science and Technology, Shenzhen, 518055, China; Department of Physics and Institute of Advanced Materials, Hong Kong Baptist University, Hong Kong, China; School of Optoelectronic Engineering and Instrumentation Science, Dalian University of Technology, Dalian 116024, China; Shenzhen Engineering Research Center for Novel Electronic Information Materials and Devices, Southern University of Science and Technology, Shenzhen 518055, China

**Keywords:** metasurface, nonlinear wavefront engineering, optical holography, orbital angular momentum

## Abstract

In linear optical processes, compact and effective wavefront shaping techniques have been developed with the artificially engineered materials and devices in the past decades. Recently, wavefront shaping of light at newly generated frequencies was also demonstrated using nonlinear photonic crystals and metasurfaces. However, the nonlinear wave-shaping devices with both high nonlinear optical efficiency and high wave shaping efficiency are difficult to realize. To circumvent this constraint, we propose the idea of metasurface decorated optical crystal to take the best aspects of both traditional nonlinear crystals and photonic metasurfaces. In the proof-of-concept experiment, we show that a silicon nitride metasurface decorated quartz crystal can be used for the wavefront shaping of the second harmonic waves generated in quartz. With this crystal-metasurface hybrid platform, the nonlinear vortex beam generation and nonlinear holography were successfully demonstrated. The proposed methodology may have important applications in nonlinear structured light generation, super-resolution imaging, and optical information processing, etc.

## 1 Introduction

Optical wavefront engineering is of critical importance in the areas of optical imaging, optical communication and information processing. In linear optics, photonic metasurface composed of artificially engineered subwavelength structures has revolutionized the technology of wavefront engineering with its unique ability to control light at the subwavelength scale [1]. An abundance of novel photonic metasurfaces, such as the metalenses, vortex beam generators, metasurface holograms, and so on [2], [3], [4], have been developed for multifunctional wavefront engineering. In nonlinear optics, wavefront engineering is also attractive because one can play with light at new wavelengths. The nonlinear wavefront engineering with traditional nonlinear crystal has been extensively studied. For example, optical phase conjugation in second or third-order processes can be used to generate time-reversal wavefront and enables correction of image aberration [5], [6], [7]. In addition, spatial light modulator (SLM) can be used to dynamically control the nonlinear waves generated from the traditional crystals, representing a hybrid way for nonlinear wavefront engineering [8, 9]. It should be noted that bulk crystals lack the ability to realize complicated nonlinear bean shaping, and the SLM is expensive and bulky. Therefore, artificially structured materials, such as the nonlinear photonic crystals and metasurfaces with engineered nonlinearity distribution have emerged as new paradigms to circumvent the above constraints.

In the design of nonlinear photonic crystals, the electric poling technique can be used to locally manipulate the optical nonlinearities. Many interesting applications, such as the generation of nonlinear Airy beams and vortex beams [10, 11], nonlinear Talbot effect [12], have been successfully demonstrated. Recent development in the laser direct writing technique was also used to make three-dimensional nonlinear photonic crystals [13, 14]. These nonlinear photonic crystals can be used for the generation of optical vortex and holographic image at second harmonic frequency [15], [16], [17], [18]. Usually, the size of the unit cell of nonlinear photonic crystal is much larger than the working wavelength of light, this will lead to high-order diffractions and low optical efficiency.

The nonlinear metasurface is a new platform for manipulating nonlinear waves with multiple degrees of freedom, including phase, amplitude, and polarization, etc. [19], [20], [21]. For example, both the dielectric and plasmonic nonlinear metasurfaces have been developed for nonlinear holography [22], [23], [24], [25], [26]. However, the frequency conversion efficiency is usually limited by the short light–matter interaction length on metasurfaces. Sustaining efforts have been devoted to circumvent this limitation. For example, multiquantum-well and epsilon-near-zero material are introduced into the design of nonlinear metal–dielectric hybrid metasurfaces [27], [28], [29].

It has been reported that the metasurface-nonlinear medium hybrid system can be used for nonlinear wavefront engineering. For example, a gold microstructure-conjugated polymer system was utilized to generate vortex beam at third harmonic frequency [30]; a metasurface-tungsten disulfide hybrid platform was developed for focusing and beam steering of the second harmonic waves (SHWs) [31, 32]. In addition, a metalens array together with a nonlinear crystal forms a high-dimensional multiphoton quantum source [33]. It can be found that in most of the previous metasurface-nonlinear media hybrid systems, the metasurface was mainly used to control the wavefront of the fundamental waves (FWs).

**Figure 1:**
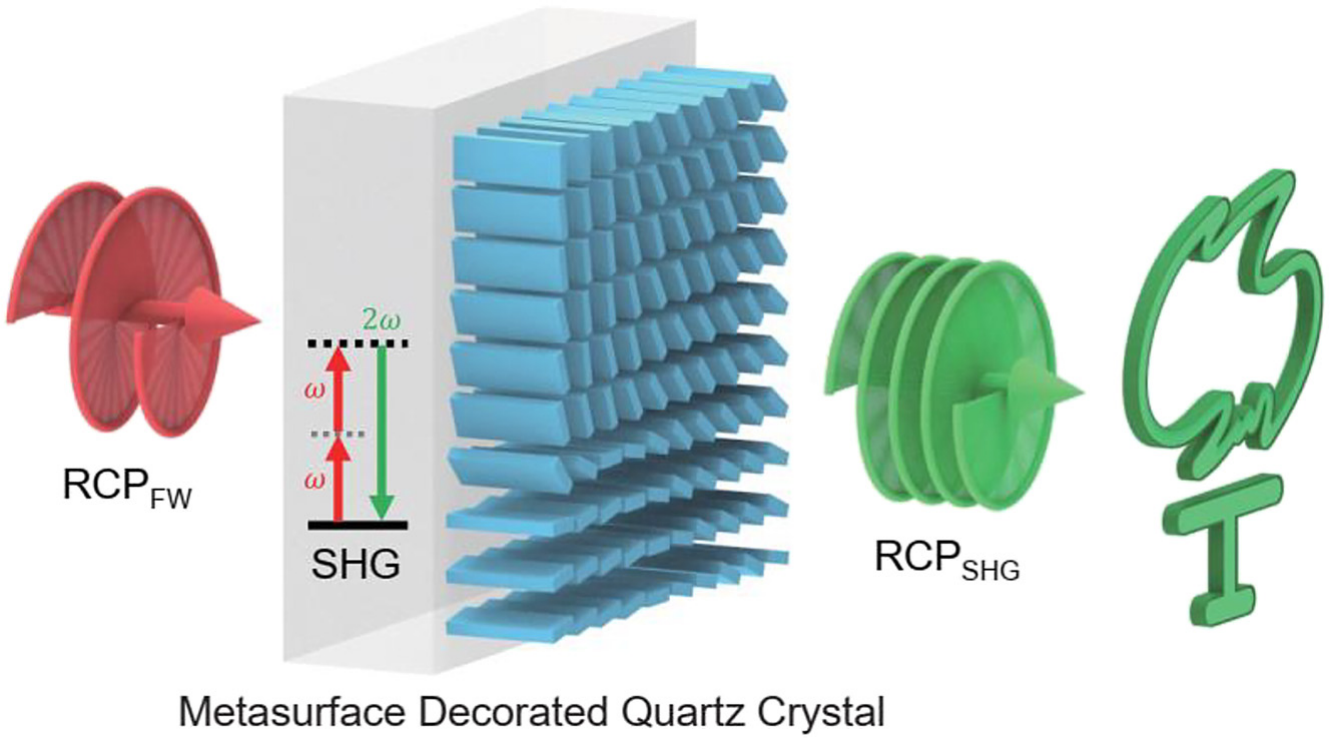
Schematic illustration of the metasurface decorated quartz crystal for nonlinear wavefront engineering. The second harmonic generation (SHG) process occurs in the quartz crystal. By integrating geometric phase controlled dielectric metasurface on top of the crystal, the wavefront engineering of the second harmonic waves will lead to a holographic image, such as generating a “Torch”. The circular polarization state of the SHW is the same as that of the fundamental wave (FW) because two circular polarization-flipping processes occur subsequently in the quartz crystal and the dielectric metasurface.

In this work, we alternatively propose the concept of metasurface decorated optical crystal to achieve compact and highly efficient nonlinear wavefront engineering. In the proof-of-concept experiment, we used a quartz crystal to generate second harmonic waves and a subsequent dielectric metasurface on top of the quartz to simultaneously control the wavefront of the second harmonic waves. As the schematic illustration shown in Figure 1, the SHWs are generated when the FW excites the quartz crystal. Then, the SHWs from the quartz pass through the dielectric metasurface made of silicon nitride (SiN_*x*_) nanofins, undergoing a phase modulation process. Finally, a holographic image at second harmonic frequency is projected to the far field. The wavefront engineering function of the metasurface is based on the geometric phase introduced by the in-plane rotations of the nanofins. To verify the proposed idea, we experimentally demonstrate nonlinear vortex beam generation and nonlinear optical holography using second harmonic waves generated from the quartz crystal. Our results indicate an easy-to-use and promising platform for realizing compact and efficient nonlinear wavefront engineering and will potentially contribute to the development of nonlinear structured light source and optical information processing, etc.

## 2 Design and fabrication of the metasurfaces

In this work, the quartz crystal with thickness of about 250 μm is used as for second harmonic generation (SHG). In the SHG process, the relationship between the induced nonlinear polarization $P^{\left(2\omega \right)}$ of a nonlinear medium and the applied optical filed $E^{\left(\omega \right)}$ is $P_{i}^{\left(2\omega \right)}=\epsilon_{0}\sum_{jk}\chi_{ijk}^{\left(2\right)}E_{j}^{\left(\omega \right)}E_{k}^{\left(\omega \right)}$ , where $\epsilon_{0}$ is the vacuum permittivity, $\chi^{\left(2\right)}$ is the second-order susceptibility tensor, and i , j , and k are indices of the Cartesian coordinates [34]. Assuming that the FW only possesses transverse components $E_{x}$ and $E_{y}$ , the nonvanishing tensor elements of the second-order susceptibility $\chi^{\left(2\right)}$ of a c-cut quartz are $\chi_{xxx}^{\left(2\right)}=-\chi_{xyy}^{\left(2\right)}=-\chi_{yyx}^{\left(2\right)}=-\chi_{yxy}^{\left(2\right)}=\chi_{1}^{\left(2\right)}$ . Therefore, when the FW is circularly polarized, i.e. $E^{\left(\omega \right)}=E_{0}{\left[\begin{array}{ll} 1 & \sigma i \end{array}\right]}^{T}$ , where σ=±1 represents the left- or right-circular polarization (LCP or RCP), the induced SHW from quartz can be derived as $P^{\left(2\omega \right)}=2\epsilon_{0}\chi_{1}^{\left(2\right)}E_{0}^{2}{\left[\begin{array}{ll} 1 & -\sigma i \end{array}\right]}^{T}$ . That is, the circular polarization states of the FW and the SHW are opposite to each other [19].

**Figure 2:**
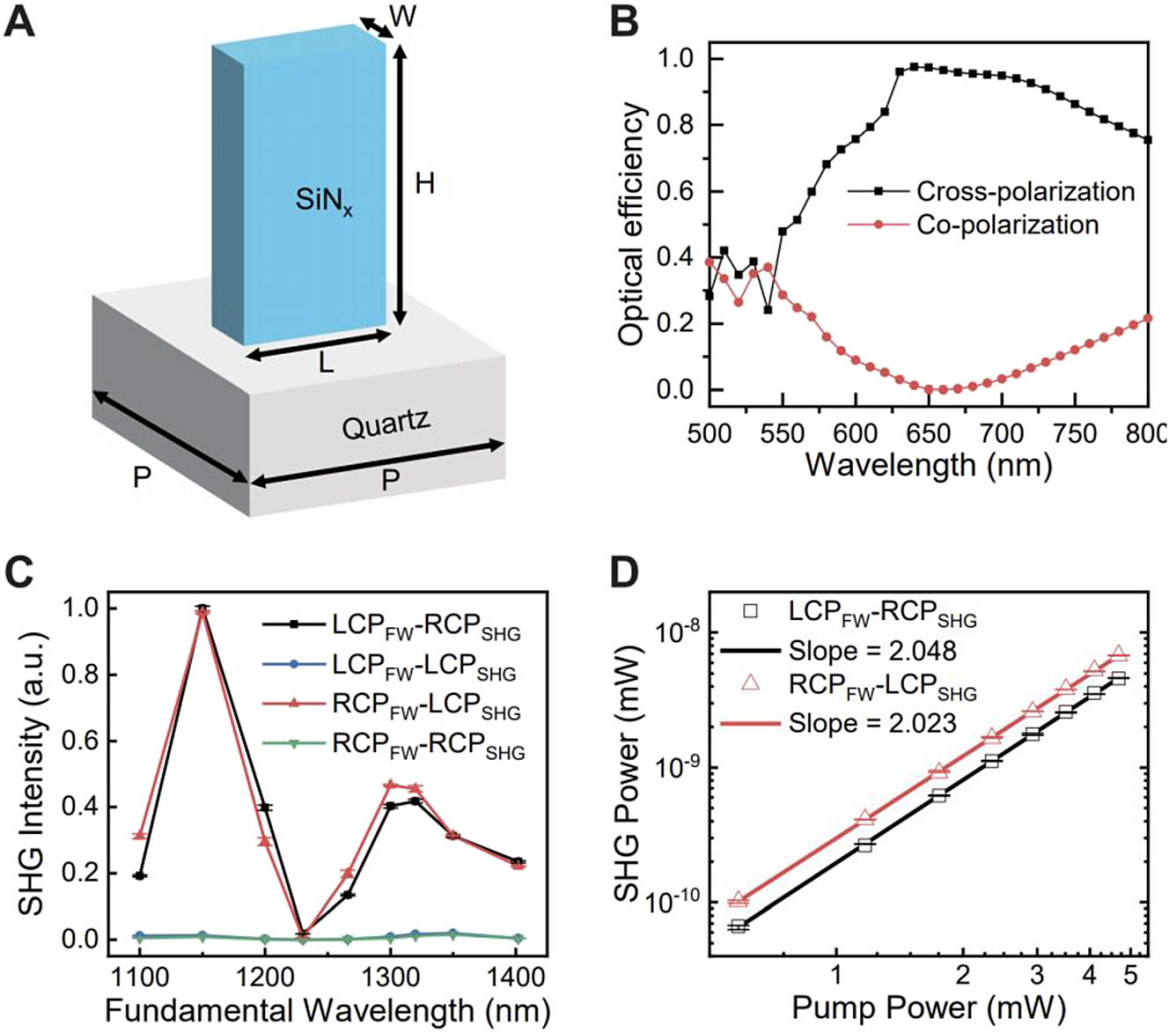
Design and linear optical properties of the dielectric metasurfaces and the nonlinear properties of the quartz crystal. (A) Schematic diagram of the silicon nitride (SiN_*x*_) nanofins, which form the dielectric metasurfaces. The structure parameters are period *P* = 430 nm, width *W* = 120 nm, length *L* = 370 nm, and height *H* = 1400 nm, respectively. (B) Numerically calculated circular polarization conversion efficiency of the metasurface. The black line indicates the cross-polarization conversion efficiency and the red line corresponds to the copolarization conversion efficiency. (C) The relative SHG intensities from the quartz crystal when the fundamental wavelength is tuned from 1100 to 1400 nm. Four curves correspond to the four circular polarization combinations of the fundamental wave (FW) and the second harmonic wave (SHG). (D) The relation between the SHG power and the pumping power at the fundamental wavelength of 1266 nm. The axes are in log-log scale and the experimental data is fitted via linear regression.

The wavefront engineering component, namely a linear photonic metasurface fabricated on the quartz crystal, consists of SiN_*x*_ nanofins working at the wavelength of 633 nm. The nanofins are birefringent and act like half-wave plates. We optimized the geometrical parameters of the nanofins by using Lumerical FDTD models. The nanofins can efficiently convert the circularly polarized SHWs into the opposite polarization state and simultaneously impart the predesigned geometric phases into the converted waves. As shown in Figure 2A, the optimized SiN_*x*_ nanofins are of height H=1400 nm, length L=370 nm, width W=120 nm, and period P=430 nm. The simulated polarization conversion efficiencies of the nanofins at wavelengths ranging from 500 to 800 nm are shown in Figure 2B. The calculated cross-polarization efficiency are higher than 90% at the target wavelength of 633 nm and exhibits a broadband high-efficiency region (>80%) spanning from 610 to 700 nm.

The realization of beam shaping function relies on the capabilities of controlling the phase of the second harmonic waves by virtue of the geometric Pancharatnam–Berry (P–B) phase [35, 36]. In the case of a nanofin, the linear P–B phase is given by ϕ=2σθ , where θ is the orientation angle, and σ represents the spin state of the incident light. Therefore, the phase profiles to achieve various optical functions can be introduced by the spatially variant nanofins. In the fabrication process, we firstly deposited a layer of SiN_*x*_ on the quartz substrate by using plasma-enhanced chemical vapor deposition technique. Then, the standard electron-beam lithography followed by a metal lift-off process was used to make a chromium hard mask. Finally, the inductively coupled plasma etching was used to make the SiN_*x*_ metasurface (see Supplementary SI-1).

Since the nonlinear frequency conversion mainly occurs in the quartz crystal, we firstly measured the wavelength dependent responses of the SHGs. In the nonlinear optical experiment, a circularly polarized femtosecond laser from an optical parameter oscillator (pulse duration ∼ 250 fs, repetition frequency 80 MHz) was used to excite the quartz crystal. The LCP and RCP components of the SHWs in the transmission direction were recorded (see Supplementary SI-2). The measured SHG responsivities of the quartz crystal are presented in Figure 2C. It clearly shows that the cross-polarization SHG signals (LCP_FW_–RCP_SHG_ and RCP_FW_–LCP_SHG_) are much stronger than the copolarization signals (LCP_FW_–LCP_SHG_ and RCP_FW_–RCP_SHG_), which is consistent with the theoretical analysis. The SHG responses at the target wavelength of 1266 nm are considered to be strong enough for the proof-of-concept demonstration. As shown in Figure 2D, we also measured the quartz crystal’s power-dependent SHG intensities at the fundamental wavelength of 1266 nm. The intensities of SHG signals are quadratic to that of the FW, a characteristic of the second-order nonlinear optical processes.

## 3 Characterizations of nonlinear wavefront engineering

Optical vortex beam generation is an important topic in wavefront engineering. A vortex beam contains a phase factor of ${\text{e}}^{il\varphi }$ , where l is the azimuthal index and φ is the in-plane azimuthal angle [37]. It plays essential role in fundamental physics because it carries an orbital angular momentum of lℏ per photon [38, 39]. Moreover, it can be used in many applications such as microscopy [40], optical tweezers [41], optical communications [42], and quantum optics [43], etc.

**Figure 3:**
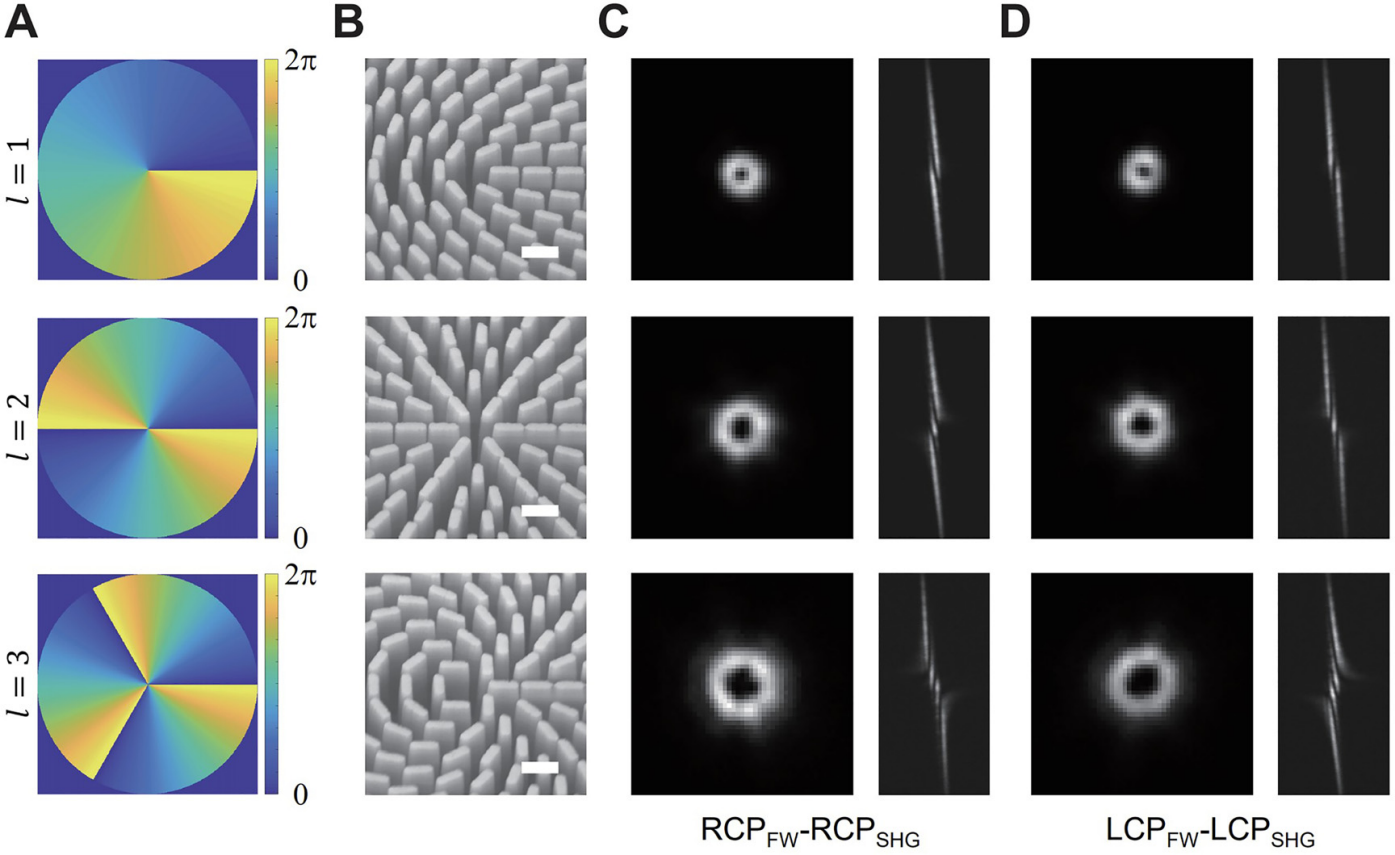
Generation of second harmonic vortex beams from the metasurface decorated quartz crystal. (A) The required phase distributions for the generation of second harmonic vortex beams with orbital angular momentum of l=1,2,3 (top to bottom). (B) The scanning electron microscope (SEM) images of the fabricated dielectric metasurfaces. Scale bars: 500 nm. (C-D) The intensity profiles of the second harmonic vortex beams imaged by using a spherical (left panel) and a cylindrical lens (right panel). The value and sign of the azimuthal index can *l* be identified from the number and orientation of the dark fringes of the cylindrical lens imaged pattern. The images of RCP_FW_-RCP_SHG_ and LCP_FW_-LCP_SHG_ measurement schemes are presented. The SHG vortex beams in these two scenarios have opposite azimuthal indices, which are determined by the circular polarization states of the FW and the design of the metasurfaces.

In the first experiment, we used a metasurface decorated quartz crystal to generate nonlinear vortex beam with orbital angular momentum values of l=1,2,3 . This is a two-step process. Firstly, the quartz crystal is used to generate the SHW with circular polarization states. Then, the SHW passes the linear optical metasurface with phase singularity. In this way, we can say that the nonlinear vortex beam was realized in the system composed of the quartz crystal and a metasurface. The required phase distributions of the SHG vortex beams are shown in Figure 3A. The spatially varied orientation angles of the SiN_*x*_ nanofins are then defined as θ(r,φ)=lφ/2 , assuming that the generated SHWs from the quartz is left circularly polarized ( σ=1 ). Since the polarization flip occurs in both the SHG generation and the wavefront shaping processes, the polarization states of the input FW and the output vortex SHW are same as each other. We designed these samples in the polar coordinate system, and the diameter of the SiN_*x*_ metasurfaces is ∼206 μm. The scanning electron microscopy (SEM) images of the fabricated metasurfaces on top of the quartz crystal are shown in Figure 3B, where the central regions of the metasurfaces are presented. The different azimuthal indices l can be easily differentiated by how fast the nanofins rotate around the sample center.

As discussed above, the metasurface devices are designed to be excited by the FWs of RCP state. The measured intensity profiles of the SHG beams of the designed polarization configuration (RCP_FW_–RCP_SHG_) are shown in Figure 3C. The SHG intensity distributions were captured by using a spherical lens (left panel) and a cylindrical lens (right panel), respectively. The increasing size of the rings reflect that the vortex beams have different azimuthal indices, whose value and sign can be identified from the number and orientation of the dark fringes of the intensity patterns imaged with a cylindrical lens. The identified orbital angular momentum of the SHG beams agrees well with the designed value of l=1,2,3 . We also measured the SHG intensity profiles of the polarization configuration LCP_FW_–LCP_SHG_, the obtained SHG vortex beams are of orbital angular momentum value of −1 , −2 , and −3 , as shown in Figure 3D. These results can be well understood because the SHWs from the quartz crystal are of RCP states ( σ=−1 ). Therefore the σ -dependent geometric phase introduced by the metasurfaces will be reversed, leading to the negative sign of l . The diffraction efficiency of the metasurfaces decorated quartz crystal was characterized in linear optical regime, using a supercontinuum laser as the light source. The measured cross-polarization diffraction efficiencies (LCP-RCP/RCP-LCP) are above 60% in the spectrum range from 600 to 680 nm (see Supplementary SI-3). The efficiency of undesired co-polarization state, corresponding to the zero order SHG spot, is at the level of 10–20% (see Supplementary SI-3).

**Figure 4:**
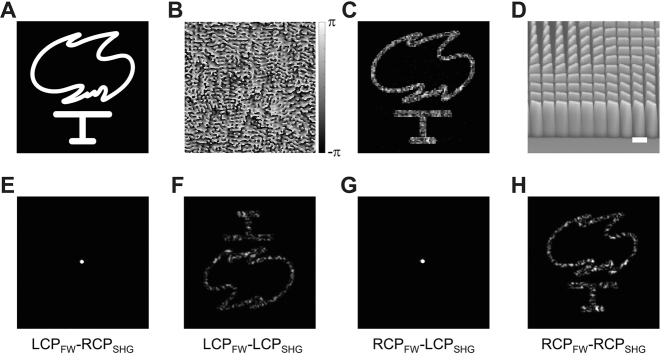
Generation of the second harmonic holographic image from the metasurface decorated quartz crystal. (A) The target “Torch” image. (B) The required phase distribution calculated by using the Gerchberg–Saxton phase retrieval algorithm. The phase hologram has 201 × 201 square pixels; each pixel size is of 430 × 430 nm^2^. The working wavelength is 633 nm. (C) Numerically reconstructed image from the phase distribution in (B). (D) The SEM image of the dielectric metasurface on top of the quartz crystal. Scale bar: 500 nm. (E–H) Experimentally recorded SHG images from the device at the fundamental wavelength of 1266 nm. Four circular polarization combinations of the FW and the second harmonic wave (SHG) are presented.

The metasurface decorated quartz crystal was further utilized to generate nonlinear holographic images. The Gerchberg–Saxton phase retrieval algorithm was used to design the phase-type hologram [44]. The target binary image and the generated phase distribution φ(x,y) are shown in Figure 4A and B, respectively. The generated φ(x,y) is based on the Cartesian coordinate system and has 481 × 481 pixels in total. The size of a unit cell of the SiN_*x*_ metasurface is 430 × 430 nm. Therefore, the overall size of the metasurface hologram is ∼206 × 206 μm. Figure 4C shows the numerically reconstructed image from φ(x,y) for working wavelength of 633 nm. The orientation distributions of the SiN_*x*_ nanofins, which are then converted to metasurface patterns for fabrication, are described by θ(x,y)=φ(x,y)/2 . The side view SEM image of the metasurface hologram is shown in Figure 4D (see Supplementary SI-4, the top view SEM image). Same as in the previous case of vortex generation, the designed polarization for the nonlinear hologram device is also RCP_FW_–RCP_SHG_. Figure 4E–H shows the recorded SHG holographic images which have four circular polarization combinations of the FW and the SHW. It can be found that the co-polarization results (LCP_FW_–LCP_SHG_ and RCP_FW_–RCP_SHG_) are the reconstructed images with qualities close to the numerical ones. The LCP_FW_–LCP_SHG_ image is rotated by an angle of 180° with respect to the target image, which is due to the fact that the geometric phase is reversed when the circular polarization of the FW is flipped. The cross-polarization results (LCP_FW_–RCP_SHG_ and RCP_FW_–LCP_SHG_) show only trivial SHG spots arising from the imperfect polarization conversion of the metasurfaces (Supplementary SI-4). Similar results can be observed in the linear holographic imaging experiment at the wavelength of 633 nm (Supplementary SI-4).

## 4 Conclusions

To summarize, we proposed the concept of metasurface decorated optical crystal as a platform for arbitrary nonlinear wavefront engineering. Based on this idea, we designed and fabricated several dielectric metasurfaces on top of the quartz crystal to demonstrate the generations of vortex beams and holographic images at the second harmonic frequency. The proposed method in this work is compatible with traditional photolithography and laser direct writing techniques, so that various crystal-metasurface hybrid systems can be designed. It is expected that the concept of metasurface decorated optical crystal may provide great potential for nonlinear wavefront engineering and optical information processing.

## Supplementary Material

Supplementary Material
