# Supplementary material for: Nonlinear wavefront engineering with metasurface decorated quartz crystal
Source: Nanophotonics. 2021 Nov 2;11(4):797–803. doi: 10.1515/nanoph-2021-0464 (PMC11501526; doi:10.1515/nanoph-2021-0464)
Supplement: Supplementary file 1 — Supplementary Material [file j_nanoph-2021-0464_suppl.docx]

Supplementary Information for

**Nonlinear** **Wavefront Engineering with** **Metasurface Decorated Quartz Crystal**

Ningbin Mao^1,2#^, Yutao Tang^1#^, Mingke Jin^1#^, Guanqing Zhang^1^, Yang Li^1,3^, Xuecai Zhang^1^, Zixian Hu^1^, Wenhao Tang^1^, Yu Chen^1^, Xuan Liu^1^, Kingfai Li^1^, Kokwai Cheah^2^ and Guixin Li^1,4*^

^1^Department of Materials Science and Engineering, Southern University of Science and Technology, Shenzhen, 518055, China.

^2^Department of Physics and Institute of Advanced Materials, Hong Kong Baptist University, Hong Kong, China.

^3^School of Optoelectronic Engineering and Instrumentation Science, Dalian University of Technology, Dalian 116024, China.

^4^Shenzhen Engineering Research Center for Novel Electronic Information Materials and Devices, Southern University of Science and Technology, Shenzhen 518055, China.

*Email: ligx@sustech.edu.cn

Table of Contents

[SI-1. Fabrication of the dielectric metasurfaces 2](#_Toc85913335)

[SI-2. Nonlinear optical response of the quartz crystal 3](#_Toc85913336)

[SI-3. Characterization of the vortex generation metasurfaces 4](#_Toc85913337)

[SI-4. Characterizations of the hologram sample 7](#_Toc85913338)

SI-1. Fabrication of the dielectric metasurfaces

Firstly, we chose a commercial double-polished quartz crystal as the substrate. The thickness of the quartz crystal is about 250 μm. In the nanofabrication process of the dielectric metasurfaces, a silicon nitride (SiN*_x_*) film of thickness about 1400 nm was grown by the plasma-enhanced chemical vapor deposition (Figure S1A). Then, the device was spin-coated with a layer of about 130 nm thick positive electron beam resist (polymethyl methacrylate, PMMA) and a layer of about 80 nm thick charge-dissipation layer, and baked on a hot plate (Figure S1B). The designed pattern is defined by the electron-beam lithography and then developed in the isopropanol diluted methyl isobutyl ketone (MIBK:IPA = 1:3) (Figure S1C). Next, a 20 nm thick chromium (Cr) hard mask is formed by using electron-beam evaporation and metal lift-off processes (Figure S1D). After that, the inductively coupled plasma (ICP) etch was used to generate the SiN*_x_* nanofins with the Cr layer as the mask (Figure S1E). At last, the Cr residual was removed by using the commercial Cr etchant (Figure S1F).


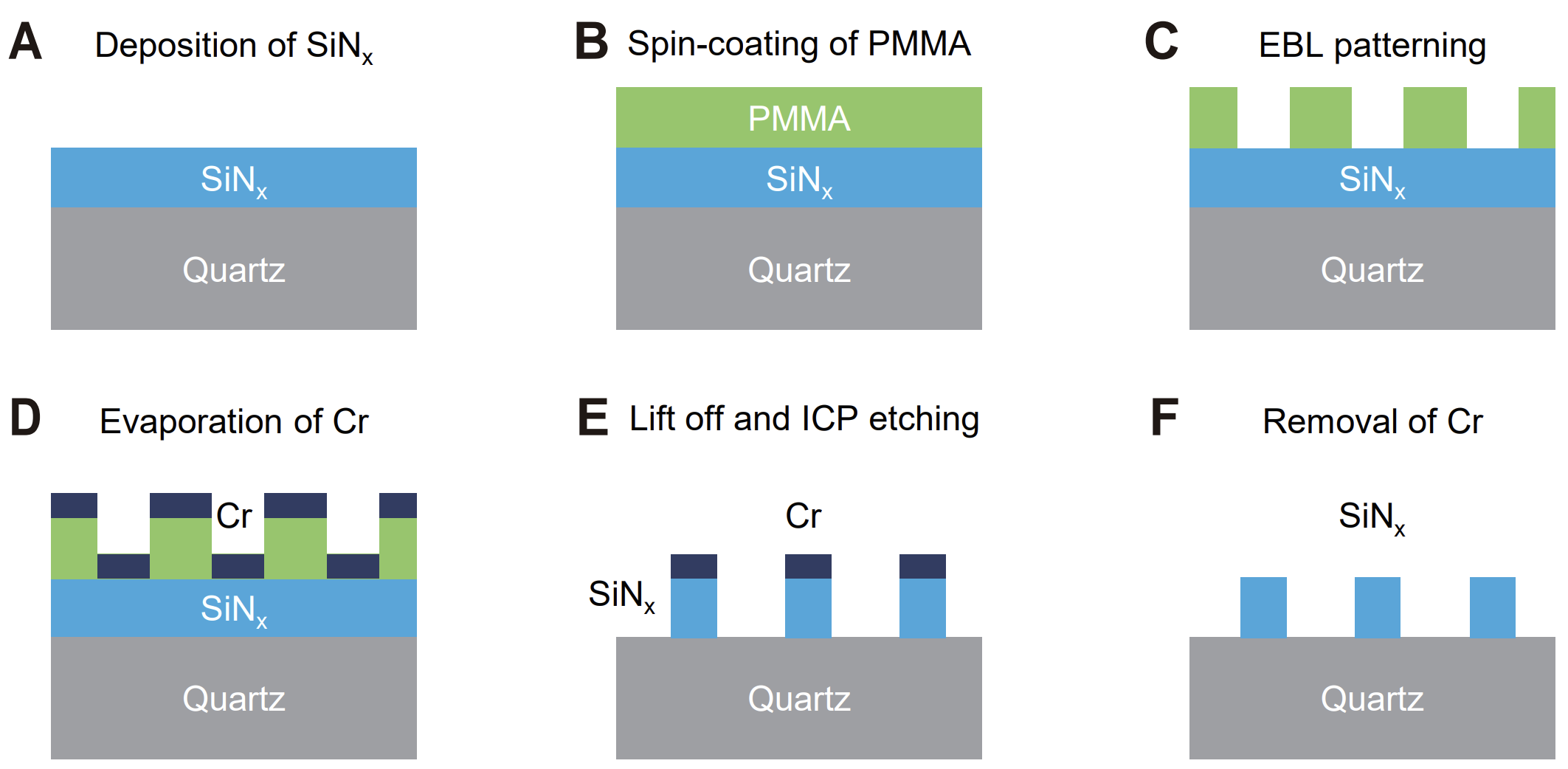


**Figure S1** Schematic illustrations of the nanofabrication process. (A) A layer of silicon nitride (SiN*_x_*) was deposited on the quartz substrate. (B) A thin film of polymethyl methacrylate (PMMA) is then spin-coated onto the SiN*_x_* thin film. (C) The electron-beam lithography (EBL) process was used to write the desired pattern into the resist layer. (D) A chromium (Cr) thin film was deposited on the patterned PMMA layer by using electron beam evaporation method. (E) The written pattern is transferred to the Cr thin film by using the lift-off process and subsequently transferred into the SiN*_x_* layer with the inductively coupled plasma etching procedure. (F) Finally, the Cr residual was removed by using the commercial Cr etchant.

SI-2. Nonlinear optical response of the quartz crystal

The second harmonic generation (SHG) signal from the quartz substrate was measured by using a femtosecond laser pumped optical parametric oscillator (pulse duration ~250 fs, repetition frequency 80 MHz). The optical setup for measuring SHG intensities in the transmission direction is shown in Figure S2. Since the maximum SHG is observed at the wavelength of 1150 nm, we also measured the power-dependent SHG intensities at the fundamental wavelength of 1150 nm (Figure S3A). The maximum frequency conversion efficiency is ~ 1.45×10^−9^ when the pumping power is 3.02 mW. As shown in Figure S3B and S3C, we also recorded the spin-resolved SHG spectra from the quartz substrate at the fundamental wavelengths of 1266 nm and 1150 nm, respectively.


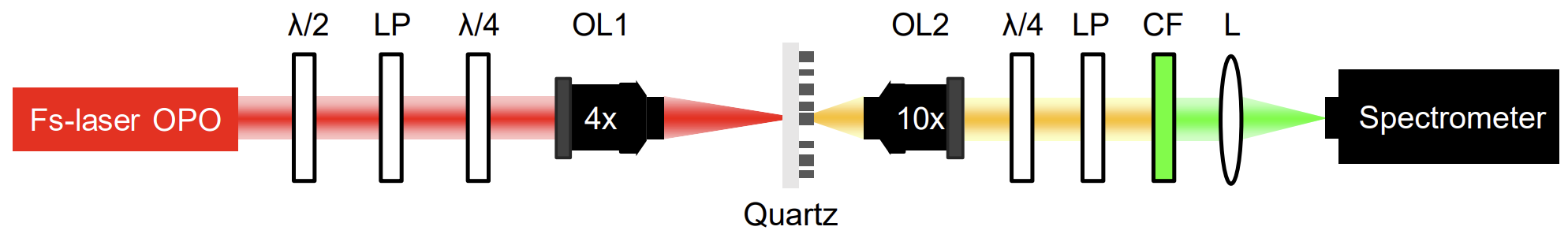


**Figure S2** Optical setup for the measurement of the second harmonic responsivities of the quartz substrate. Fs-laser OPO: femtosecond laser pumped optical parametric oscillator, λ/2: half-wave plate, LP: linear polarizer, λ/4: quarter-wave plate, OL1/OL2: objective lens (OL1: 4×, NA = 0.1 for the focus of FW; OL2: 10×, NA = 0.25 for collection of the SHG), CF: short pass color filter, L: lens.


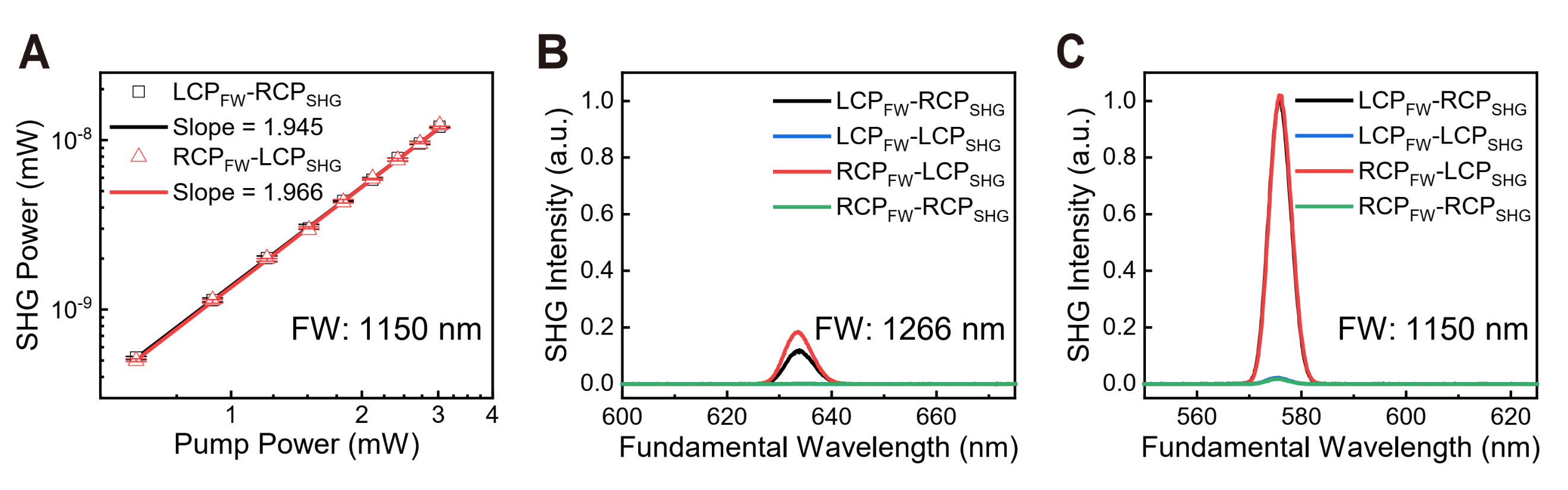


**Figure S3** (A) Measured power-dependent SHG intensities from the quartz crystal at the fundamental wavelength of 1150 nm. (B, C) The spin-resolved SHG spectra were recorded by using the spectrometer when the fundamental wavelengths are 1150 nm and 1266 nm, respectively. The SHG spectra in B and C are normalized without affecting the relative intensities.

SI-3. Characterization of the vortex generation metasurfaces

Figure S4 shows the top-view scanning electron microscopy (SEM) images of the metasurface samples for vortex beam generation. At the center of the SEM images, one can easily distinguish different topological charges of the metasurface designs. The optical setup used to measure the intensity profiles of the SHG vortex beams is illustrated in Figure S5. The intensity profiles of the SHG vortex beam imaged by using a spherical lens are shown in Figure S6, where four circular polarization combinations of the FW and SHW were used. As mentioned in the main text, we measured the linear optical properties of the metasurface devices by using a supercontinuum laser. The linear optical measurement setup is shown in Figure S7, and the measured optical efficiencies are shown in Figure S8. In linear optical regime, we also measured the intensity profiles of the vortex beams generated at the wavelength of 633 nm. The results corresponding to four circular polarization combinations of the incident and analyzed light waves are shown in Figure S9.


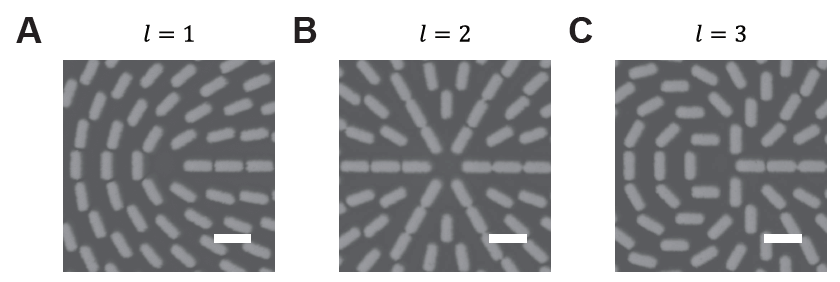


**Figure S4** (A-C) Top-view scanning electron microscopy (SEM) images of the metasurfaces for generation of vortex beam with orbital angular momentum values of $l=$1$,2, 3$. It is easy to find that the samples are designed in the polar coordinate system. These images were taken at the central area of each device so that the topological charge of the SiN*_x_* metasurface can be identified. Scale bar: 500 nm.


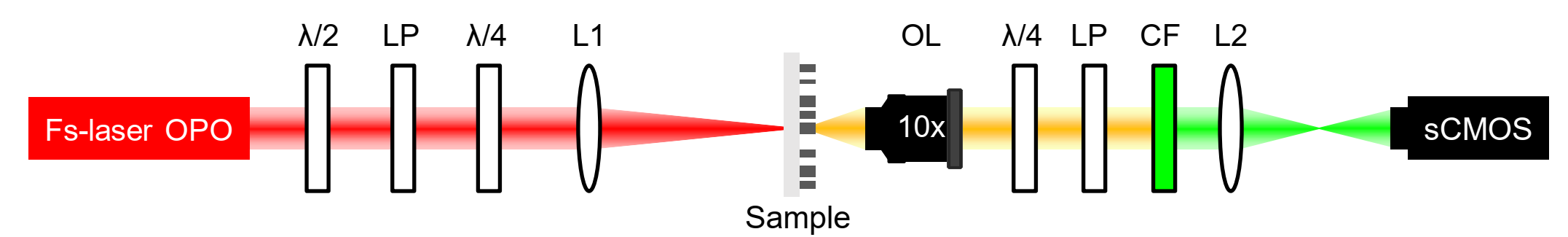


**Figure S5** Optical setup for measuring the intensity profiles of the SHG vortex beams. The FW is focused using a long focal lens (L1, *f* = 250 mm). The diameter of the laser spot is about 100 μm, which is close to the sample size (206.4 μm in diameter). The SHG signals were collected in the transmission direction by using an objective lens (OL, 10×, NA = 0.25). After passing through the polarization analyzer and the color filter, the intensity profiles of the second harmonic waves were projected onto a sCOMS camera by using a spherical or cylindrical lens (L2, the focal lengths of the spherical and the cylindrical lens are 100 mm and 75 mm, respectively).


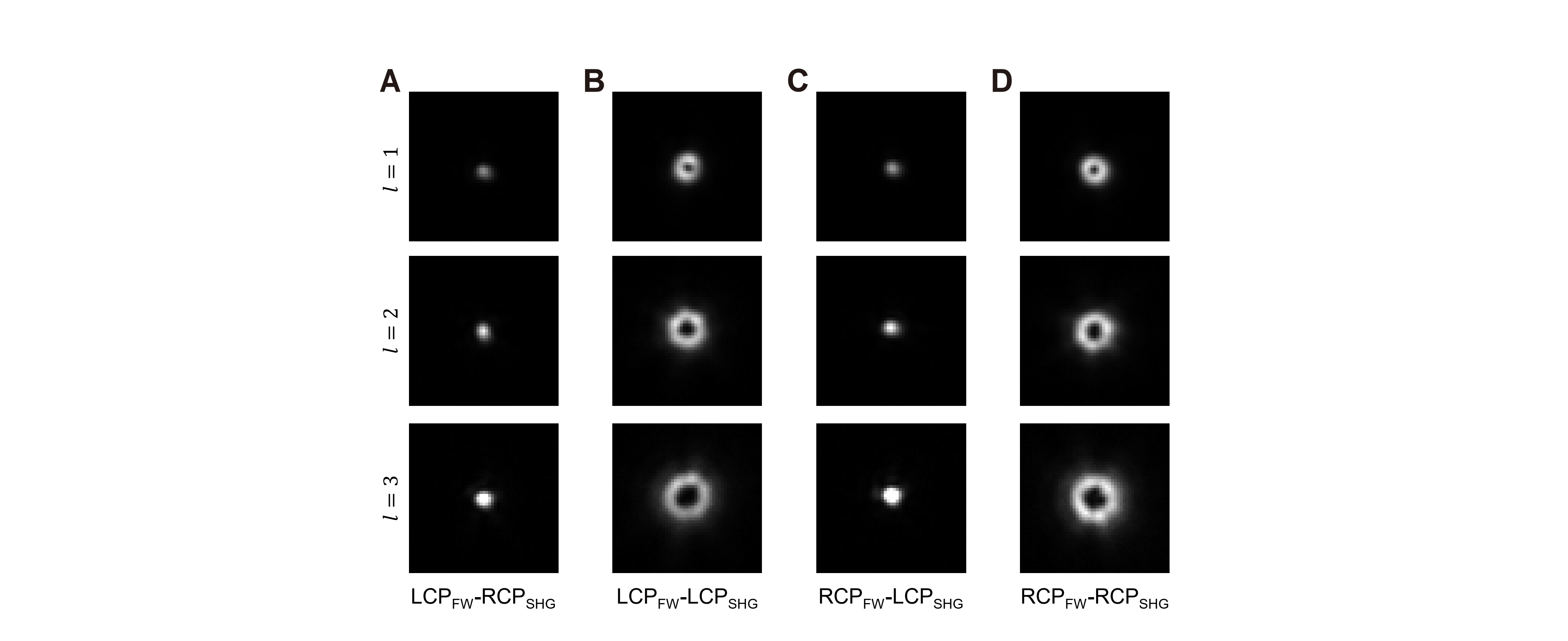


**Figure S6** Intensity profiles of the SHG vortex beams imaged by using the spherical lens. The fundamental wavelength is 1266 nm. The four circular polarization combinations of the FW and SHW are presented. The co-polarization results in B and D are same as that in Figure 3C and 3D of the main text.


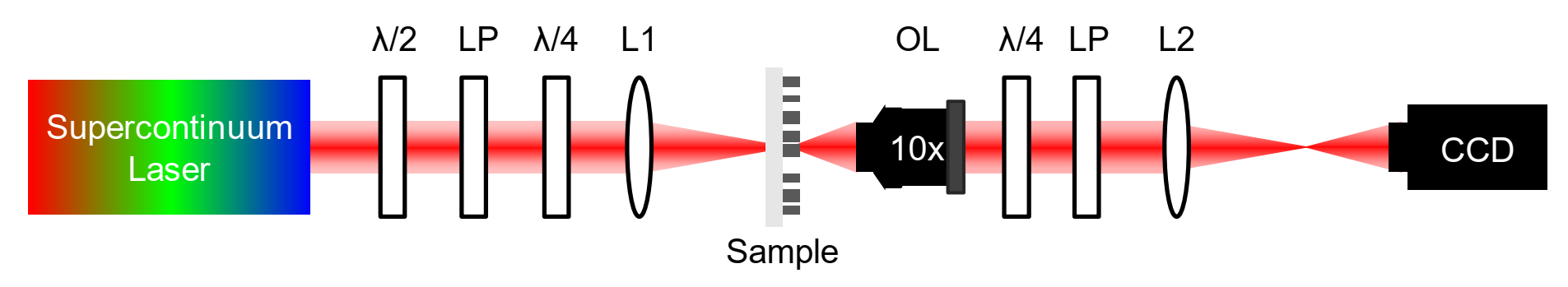


**Figure S7** Optical setup for measuring the linear properties of the metasurface decorated optical crystal. A supercontinuum laser is used as the light source. λ/2: half-wave plate, LP: linear polarizer, λ/4: quarter-wave plate, L1: focus lens, OL, objective lens (10×, NA = 0.25), L2: tube lens (spherical or cylindrical). We used a lens with focal length (*f* = 200 mm) as L1 when taking the intensity profiles. When measuring the optical efficiency of the devices, a lens with focal length *f* = 100 mm was used. The power detector is placed behind L1 and L2 to measure the input and output power of light.


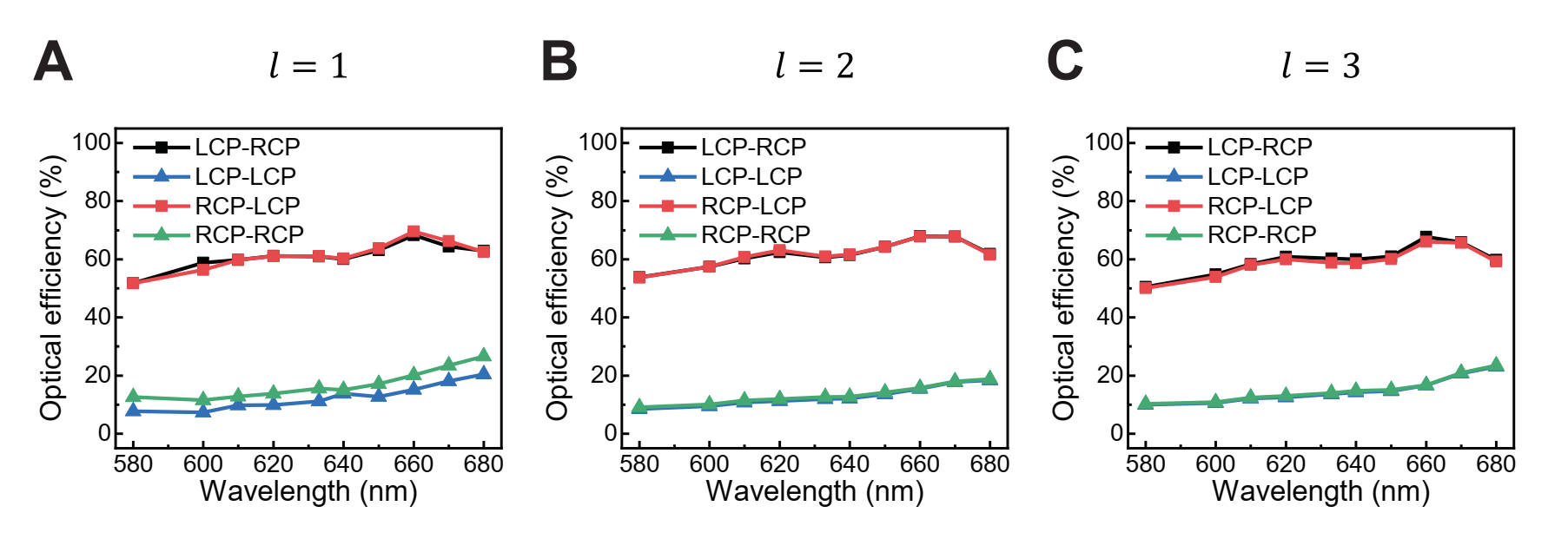


**Figure S8** Measured linear optical efficiencies of metasurfaces for vortex beam generation. Four curves correspond to four circular polarization combinations of the incident and transmitted light. The efficiency for the cross-polarization combination (LCP-RCP and RCP-LCP) are above 50% across the measured spectral range, and the efficiency for co-polarization combination (LCP-LCP and RCP-RCP) is much lower, contributing to the zeroth-order SHG spots in Figures S6A and S6C.


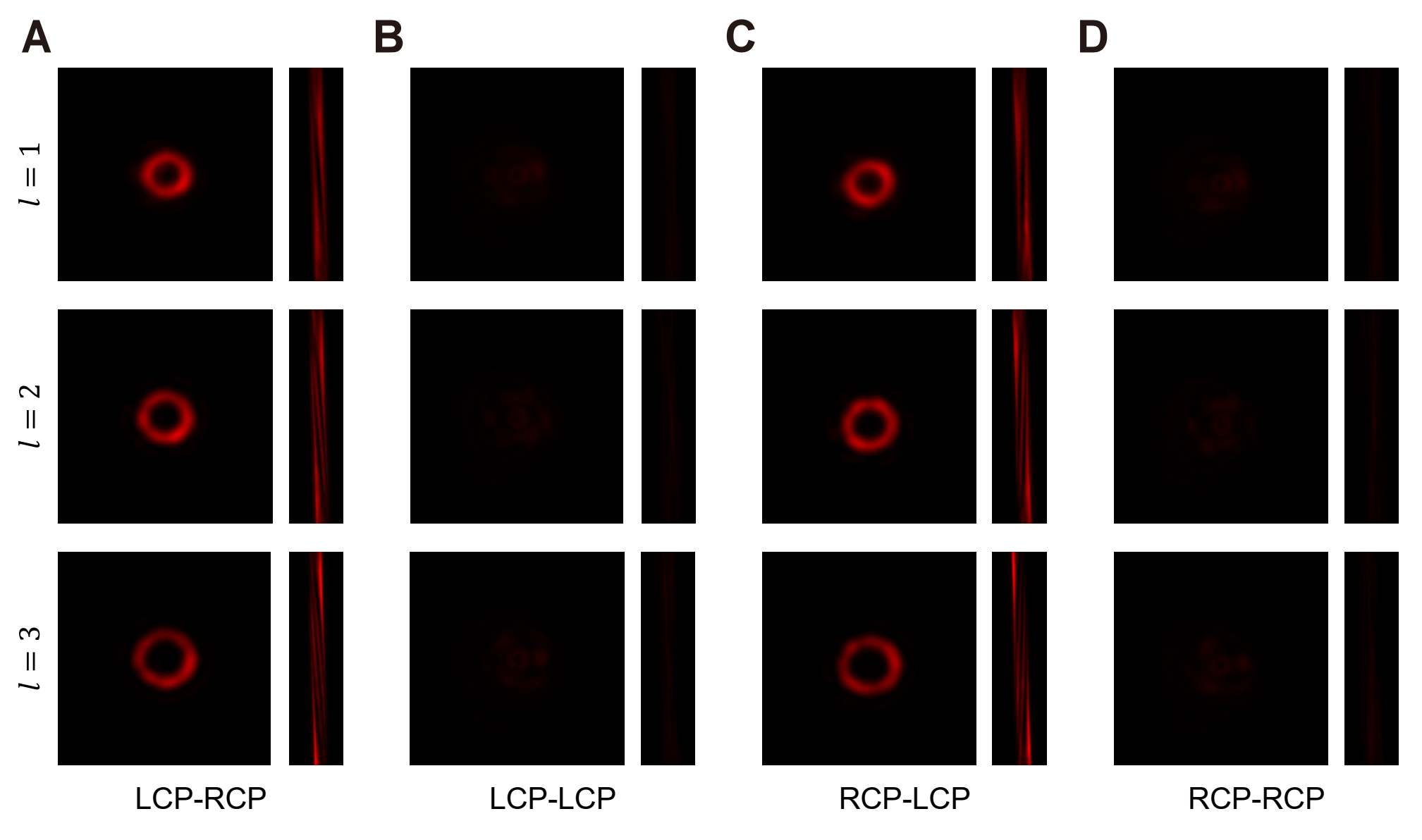


**Figure S9** Measured intensity profiles of vortex beam generated from the metasurface. The results corresponding to the four circular polarization combinations of the input and output light waves are recorded at wavelength of 633 nm. For each polarization combination, the intensity profiles imaged by using the spherical lens (left) and the cylindrical lens (right) are presented. The orbital angular momentum values of the vortex beams can be identified from the fringe patterns, which are of opposite values for LCP-RCP and RCP-LCP polarization combination.

SI-4. Characterizations of the hologram sample

Figure S10 shows the top view SEM image of the metasurface (Figure S10A) and the measured linear optical efficiency (Figure S10B). The hologram sample’s cross- and co-polarization diffraction efficiency are at the range of 50% ~ 60% and 5% ~ 20% for incident wavelength ranging from 580 nm to 680 nm. Figure S11 shows the linear optical holographic images at wavelength of 633 nm. The cross-polarization results (LCP-RCP and RCP-LCP) are the reconstructed images. The co-polarization ones (LCP-LCP and RCP-RCP) are the zeroth-order spots arising from the imperfect polarization conversion of the metasurfaces.


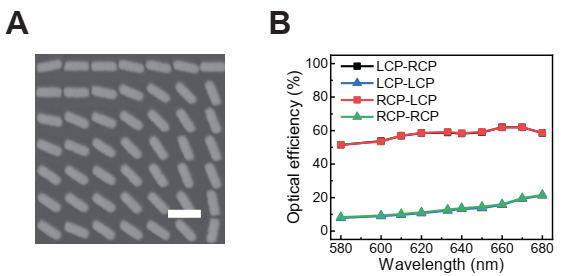


**Figure S10** Top-view SEM image of the metasurface hologram and the measured linear optical efficiency. (A) Top-view SEM image. The sample is designed in the Cartesian coordinate system. Scale bar: 500 nm. (B) Measured linear optical efficiency of the metasurface hologram. The optical setup used for the measurement is shown in Figure S7.


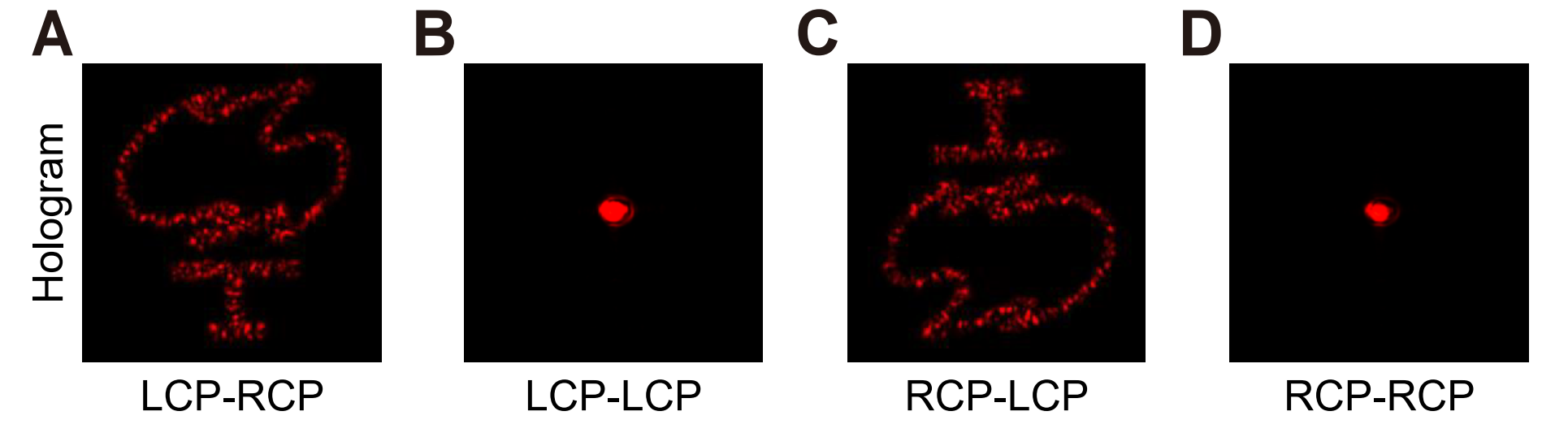


**Figure S11** Experimentally recorded linear optical holographic images from the metasurface hologram. The results correspond to four circular polarization combinations of the input and output light at the wavelength of 633 nm. The optical setup used for the measurement is shown in Figure S7.
